# Compassionate and Proactive Interventions by Health Workers in the United Kingdom: A Better Approach to Prevent and Respond to Female Genital Mutilation?

**DOI:** 10.1371/journal.pmed.1001982

**Published:** 2016-03-22

**Authors:** Maria Luisa Amasanti, Mendinaro Imcha, Comfort Momoh

**Affiliations:** 1 Acute Internal Medicine, Royal Free London NHS Foundation Trust, London, United Kingdom; 2 Obstetrics & Gynaecology, University Hospital Limerick, Limerick, Ireland; 3 African Well Woman’s Clinic/Midwifery Department, Guy’s and St Thomas’ NHS Foundation Trust, London, United Kingdom

## Abstract

Amasanti and colleagues consider female genital mutilation in the UK, how overly intrusive efforts to help might make the problem worse, and how best to move forwards.

**Summary Points**

- The United Kingdom government wants to reduce the prevalence of female genital mutilation (FGM) in the country. In order to do this, the UK government has introduced mandatory reporting of all FGM detected as child abuse.
- Is this the best first-line approach, or could it drive the problem even further underground or inadvertently harm those who have suffered from FGM?
- A more effective first-line intervention to reduce the prevalence of FGM could be a 3-fold approach comprising the following: (1) educating and training health workers about FGM, (2) trained health workers offering information about FGM to females at risk, and (3) antenatal screening.
- We hope that by implementing these interventions, it would reduce the necessity for all FGM detected to be mandatorily reported as child abuse.
- There is the possibility that these three steps may not be enough and that some form of monitoring of females at risk of FGM may need to be considered. We look at the issues this raises.

## Background

Female genital mutilation (FGM) has been illegal in the UK since 1985. Since 2003, anyone found guilty of an offence under the Female Genital Mutilation Act is liable to a prison sentence of up to 14 years [1]. Data published in 2014 state that the number of women and girls in England and Wales at risk of or already with FGM has increased significantly [2]. The study used data from birth registrations and the 2011 census to determine demographic data about women born in countries where FGM is practiced and girls born to them. The study states that in 2011, an estimated 137,000 women who had suffered FGM were living in England and Wales. Compared to data collected in 2007 [3], this shows that the prevalence of FGM in the last 7 years has increased by approximately 43%. Despite this increase, a select committee report in 2010 [4] documented that the Metropolitan Police Service had only been involved with 145 “incidents of concern” relating to FGM between 2008 and 2011. It is as yet unclear as to why the prevalence of FGM appears to have risen without a corresponding increase in convictions, although there are a number of possible reasons to consider. Firstly, since FGM has been a priority on the public agenda, more women who have been victims of FGM may have come forward because they feel it is now safe to do so. In addition, more research and data gathering on FGM is now in place than ever before, making our estimates of prevalence more accurate. We also need to take into account new female immigrants who are now in the UK and have already been victim to FGM in their countries of origin and recognize that in such instances FGM is happening somewhere else and not in the UK.

The UK Government's Every Child Matters: Change for Children Programme states that all agencies have a responsibility to keep children safe and protect them from harm [5]. Adding this condition to the FGM legislation of 2003, health workers today have a statutory responsibility to safeguard children from being abused through FGM [6]. There are a further two international conventions under which the UK and other signatory states also have an obligation to take legal action against FGM [7].

The question then asked is, why have there been so few incidents of FGM reported by health workers in the UK? According to the London Safeguarding Children Board [7] and the Home Office [8], the most common reasons appear to be the following:

- One or both parties finding the subject of FGM difficult and uncomfortable
- Fear of offending the woman
- Health workers being unsure of what to do/say
- The woman’s reluctance to be identified as a victim of FGM

As FGM is now on the public agenda, significant changes have also been introduced for health workers by the UK government. The onus is on them to report any cases that they come across. For example, since April 2014, National Health Service (NHS) hospitals have been required to record if a patient has had FGM, if there is a family history of FGM, or if an FGM-related procedure has been carried out on a woman, while from September 2014, all acute hospitals must report the number of patients with FGM centrally to the Department of Health on a monthly basis [9]. Submission also became mandatory for general practitioners (GPs) and NHS mental health trusts from October 2015. These data have subsequently been reported quarterly by the Health and Social Care Information Centre (HSCIC). The most recent report for the quarter from July to September 2015 showed the following:

- There were 1,385 newly recorded cases of FGM reported, with 1,641 total attendances in which FGM was identified or a procedure for FGM was undertaken.
- Over 50% of women and girls in both the newly recorded and total attendance cohorts lived in the Greater London region.
- There were 17 women or girls under the age of 18 at the point of first attendance, 1.2% of newly recorded cases.
- Self-report was the most common FGM identification method, accounting for 71.1% of newly recorded cases in which the means of FGM identification was known.
- Eight newly recorded women or girls were reported to have been born in the UK [10].

Mandatory data recording is a useful intervention as it will provide us with more accurate information, enabling more informed decisions to be made and acted upon.

Since October 2015, regulated health and social care professionals and teachers in England and Wales have a mandatory requirement to report visually confirmed or verbally disclosed cases of FGM in girls under the age of 18 to the police [11]. Mandatory reporting of all FGM as child abuse is a point of contention with hugely disparate opinions about whether this would be an effective solution. There is a fear by some public bodies that mandatory reporting of FGM as child abuse and the threat of prosecution could potentially drive the problem of FGM even further underground [12]. Furthermore, since it is often a close family member who is the instigator or perpetrator, legal action leads to “double victimization” of the child [13], and prosecution and imprisonment of her parents may often not be in her best interests. It has also been noted that some women with FGM are recent immigrants to the UK and lack a confirmed immigration status. These women may be afraid that involvement with any statutory agency will lead to deportation, and for that reason, a woman may prefer to compromise her health [14].

As clinicians working in relevant specialities, we come across FGM on a regular basis. In our experience to date, we do not believe that the introduction of mandatory reporting of FGM as child abuse is the most appropriate first measure. Rather, we believe that three key steps should have come before this, which, if implemented, could help towards reducing the prevalence of FGM in the UK. The three steps are as follows:

1. Training health workers in relevant specialties in the issues surrounding FGM
2. Trained health workers educating at-risk females about FGM
3. Incorporating mandatory screening for FGM risk factors during antenatal care

## FGM Education and Training for Health Professionals

As health workers, we want to make our patients feel safe enough to come forward and seek help and/or treatment from us. There are at present 15 FGM clinics in England [15] that women can attend for deinfibulation (a small procedure to open the scar), support, and information. There are not yet any clinics in Wales or Scotland [16]. We hope that the women who have already attended these clinics have felt supported and safe enough to encourage their peers to attend and that making an appointment at an FGM clinic will soon lose any stigma that may be attached to it. The most recent figures from the Department of Health cited above would seem to support the current approach in FGM clinics as an effective one, as over 70% of new cases of FGM identified were through women self-reporting. However, this still implies that approximately 30% do not wish or feel able to seek support. If our patients are deterred from approaching us in the first place because of fear of the repercussions, then mandatory reporting to the police becomes a hindrance rather than a help, and furthermore, it could estrange health care workers as they are prevented from caring for their patients.

The way to engage and unite health workers and patients is through education and training. Currently, some health workers still lack knowledge about FGM. We hope that through education and training, health professionals will feel more confident and equipped to work with patients affected by FGM. Furthermore, they will influence the consultation in a positive and meaningful way. A positive outcome would be that the health worker is able to communicate knowledgably and effectively about FGM, allowing the woman to feel comfortable enough to also talk about it.

Other positive changes could be as follows:

1. The woman is able to seek ongoing support and protection that prevents her from becoming a victim of FGM;
2. The woman decides to attend an FGM clinic;
3. The woman decides not to put her own daughter(s) through FGM despite a long family tradition;
4. The woman asks for a counselling referral.

As health workers in relevant specialties, we need to be educated on all aspects of FGM in order to bring about positive change. If education and training were introduced into the curriculum for those health professionals most likely to encounter FGM, such as midwives, GPs, obstetricians and gynaecologists, paediatricians, public health nurses, and doctors, we believe it could have a positive effect. This could be measured to demonstrate effectiveness.

A teaching module on FGM could be developed for the NHS Continued Professional Development Programme and be open to any health professional who wishes to attend. Following proper training, any health professional should be able to talk knowledgeably and empathetically about FGM and, with sufficient skill, to ease the taboo of discussing FGM. Health workers should be sensitive to the intimate nature of the subject and yet talk at ease about FGM, thus making the woman feel safe and free to talk. They should be able to ask straightforward questions whilst being sensitive to language barriers. They should be able to give a clear explanation about why FGM is illegal in the UK and explain how the law can be used to help the family avoid FGM if/when they have daughters. Paediatricians in particular need to use terminology that a child will understand and be sensitive to the fact that the child will be loyal to her parents. They need to give the child time to talk and obtain accurate information about the urgency of the situation. They also need to ensure that the child feels safe and secure in their presence and ensure that the child understands that the health worker is there to help her and protect her and that she can access this help and protection at any time. All health workers need to be sensitive to the mental health of patients and know how to access counselling.

Health workers’ jobs can also be facilitated by communication aids: female interpreters help reduce misunderstanding and increase the likelihood of identification of FGM, although using family members as interpreters is not advised. Health workers should also be provided with communication aids for consultations when an interpreter is not available. For example, the terms “FGM” or “cutting” are still not always understood by individuals in the practicing communities, largely because they are English terms [16]. Having flash cards available with the appropriate translations (e.g.,"Guddniin" in Somali and “Tahur” in Sudanese) could greatly facilitate a consultation.

Upon completion of training, health workers of any level should also know the risk factors and warning signs to look out for indicating that a child may be at risk of or has already undergone FGM. If a health worker does identify a female living with or at risk of FGM, it is crucial that s/he knows exactly what to do with that information and that a clear framework is available detailing exactly what services can be accessed [17]. Indeed, during the writing of this paper, Health Education England has launched an e-learning programme on FGM [18] that covers an introduction to FGM, communication skills for FGM consultations, legal and safeguarding issues, and presentation and management of FGM in young females and around pregnancy. We look forward with hope to seeing the positive effect of this in the future.

## Mandatory Screening of FGM during Antenatal Care

The Royal College of Obstetricians and Gynaecologists (RCOG) proposed antenatal screening for FGM in 2009 [19] and recently released their second edition Green Top Guideline on FGM and its management [20]. Given that daughters of mothers with FGM are at the greatest risk of being forced to have FGM themselves [21], we believe that antenatal screening for FGM is crucial for detection, intervention, and prevention. It is through mandatory antenatal screening and postpartum monitoring of female children that we can safeguard today's female children and eradicate FGM from the UK.

We propose that all antenatal booking notes be amended to contain an FGM screening section. By introducing FGM screening as a normal part of antenatal booking, awareness of FGM would be raised across the UK in a nonsensational way. Secondly, we hope this would help towards removing the taboo of FGM, as no woman who has undergone FGM in the past should have to be subject to judgmental treatment in a health care setting. The screening proposed by the RCOG was not invasive. In 2009, they suggested it should be based on family-origin questions already included to screen for potential haemoglobinopathies. In 2015, the proposal was revised to suggest that “all women, irrespective of country of origin, should be asked for a history of FGM at their booking antenatal visit.”

Any pregnant woman with FGM would then receive support, information, and counselling from a trained midwife regarding FGM. If that woman gives birth to a female child, all discussions about FGM must be documented in the discharge summary and child health record held by the parents. The health visitor and GP would then reinforce the message on education and ensure that appropriate care and support are provided.

## Monitoring of At-Risk Female Children

We hope that these steps described above would be effective, but there is of course the possibility that in some cases they will not be enough. The question is, how will we know if these steps are effective? How will we gather evidence to inform us about whether we are taking the right steps? How do we ensure that girls at risk do not become victims? This leads us to the debate of the monitoring of “at risk” girls. The definition of “at risk” girls is females from a country of origin in which FGM is commonly practiced.

If the mandatory antenatal screening tool is introduced, all female children who are at risk will be identified from birth. Exactly how these girls will then be monitored is still under debate. The UK government is in the process of introducing a pilot risk assessment tool for health workers to use with women and girls that stratifies risk (moderate, significant, or immediate) through ticking yes or no to a number of indicators that could lead to concern [17]. If concern was identified, the health care worker would then act in accordance with local safeguarding procedures and refer the case to the relevant body (police, social services, etc.). This leads us to ask the following questions:

- On which girls would the assessment tool be used?
- How would those girls be selected?
- If a girl was worried about repercussions or was being intimidated, could she manipulate the score?
- How often would this tool be used on one girl?

The idea of stratifying risk is valid, but the answers to these questions remain unclear. Fundamentally, the only definitive way to know whether a girl has undergone FGM is by physical examination, but as yet, this has not been overtly discussed in safeguarding procedures or on a public platform. Introducing compulsory physical examination will undoubtedly face great opposition, but would its potential benefit outweigh the difficulties? Should it be considered as a final recourse when a girl is stratified as being at significant or immediate risk?

How to monitor girls is a question that requires further consideration and input from all disciplines involved in order to find an option that effectively prevents abuse, respects individual rights, and is widely acceptable.

## Conclusion

FGM intervention in other parts of Europe has strongly indicated the composite nature of the issue [22]. For FGM projects to have the best chance of success, they need to be multifaceted and involve all social groups, men, women, children, health workers, community leaders, and local authorities. The scope of this paper is much narrower and focuses specifically on what can be done in the medical field. While we have already discussed the complexity of finding a suitable way to monitor girls at high risk of FGM, we believe that the three-step approach of (1) educating health workers, (2) educating women and girls at risk, and (3) incorporating mandatory antenatal screening is an effective approach that is compassionate. These are proactive steps that rely heavily upon awareness and training, along with the correct documentation and communication flowing from antenatal care to obstetric care to postpartum care to GP care. This is where the role of health workers comes to the fore and where we can make a positive change. Follow-up studies would need to be designed and conducted that were capable of assessing the results of these methods and helpful in designing improvements.

## Abbreviations

FGM: female genital mutilation
GP: general practitioner
HSCIC: Health and Social Care Information Centre
NHS: National Health Service
RCOG: Royal College of Obstetricians and Gynaecologists
